# Predictive screening for regulators of conserved functional gene modules (gene batteries) in mammals

**DOI:** 10.1186/1471-2164-6-68

**Published:** 2005-05-09

**Authors:** Sven Nelander, Erik Larsson, Erik Kristiansson, Robert Månsson, Olle Nerman, Mikael Sigvardsson, Petter Mostad, Per Lindahl

**Affiliations:** 1 Sahlgrenska Academy, Department of medical and physiological biochemistry Box 440, SE-405 30 Göteborg, Sweden; 2 Chalmers Technical University, Department of mathematical statistics, Eklandagatan 76, SE-412 96 Göteborg, Sweden; 3 Lund Strategic Research Center for Stem Cell Biology and Cell Therapy, BMC B10, Klinikgatan 26, SE-221 48 Lund, Sweden

## Abstract

**Background:**

The expression of *gene batteries*, genomic units of functionally linked genes which are activated by similar sets of cis- and trans-acting regulators, has been proposed as a major determinant of cell specialization in metazoans. We developed a predictive procedure to screen the mouse and human genomes and transcriptomes for cases of gene-battery-like regulation.

**Results:**

In a screen that covered ~40 per cent of all annotated protein-coding genes, we identified 21 co-expressed gene clusters with statistically supported sharing of cis-regulatory sequence elements. 66 predicted cases of over-represented transcription factor binding motifs were validated against the literature and fell into three categories: (i) previously described cases of gene battery-like regulation, (ii) previously unreported cases of gene battery-like regulation with some support in a limited number of genes, and (iii) predicted cases that currently lack experimental support. The novel predictions include for example Sox 17 and RFX transcription factor binding sites that were detected in ~10% of all testis specific genes, and HNF-1 and 4 binding sites that were detected in ~30% of all kidney specific genes respectively. The results are publicly available at .

**Conclusion:**

21 co-expressed gene clusters were enriched for a total of 66 shared cis-regulatory sequence elements. A majority of these predictions represent novel cases of potential co-regulation of functionally coupled proteins. Critical technical parameters were evaluated, and the results and the methods provide a valuable resource for future experimental design.

## Background

To understand how gene expression is coordinated to produce hundreds of cell phenotypes from an identical complement of genes is a principal challenge in mammalian genome research. A commonly suggested model for terminal differentiation in metazoans is that the core features of the cellular phenotype are mediated by a set of genes that is regulated as a *gene battery*, i.e. a set of functionally coupled genes that are activated by similar cis- and trans-acting regulators [1-3]. Although the gene battery is an idealized concept, concrete examples of gene battery-like regulation have been found in for example muscle subtypes [4-7], megakaryocytes [8], the epidermis [9] and lymphocytes [10,11].

A key step in the elucidation of gene battery-like regulation is to detect and functionally test the *cis *regulatory elements that mediate the co-regulation. A number of computation-based methods have been proposed to do this. In micro-organisms, computational methods have proven useful to detect modules of co-regulated genes [12,13]. In mammals, predictive models based on assumed co-regulation at the *cis *level have been constructed for liver- and skeletal muscle-selective gene regulation [14,15], and general tools have been developed for the regulatory analysis of co-expressed genes [16-18].

The aim of this work is to screen the mouse and human genomes and transcriptomes for instances where sharing of *cis*-regulatory sequences is statistically coupled to conserved co-expression of genes, i.e. cases that fall within or near the idealized gene battery concept. Another aim is to critically investigate technical parameters in order to maximize the sensitivity by which co-regulation of co-expressed genes can be detected. In a screen that covered ~40 per cent of all protein-coding genes according to the latest Ensembl annotation, we identified 21 co-expressed gene clusters with 66 cases of statistically supported sharing of cis-regulatory sequence motifs. The predictive value of the assignment of transcription factor binding sites was experimentally evaluated on EBF binding sites in a set of B-cell expressed gene clusters. The predicted cases of co-regulation include several previously known prototype examples of tissue specific regulation, but also novel predictions. All data are made available to the research community in the form of an internet resource that may serve as a starting point for further analysis.

## Results

The analysis was based on the assumption that homologous genes in mouse and human are equivalent in most aspects of regulation and function. In particular, we assumed that the transcriptional regulation is conserved for orthologous genes. For example, the mouse gene *Myh1 *and the human gene *MYH1 *are assumed to share expression pattern and to share important cis-regulatory sequences. Below, the term 'ortholog pair' will be applied as a two-species equivalent of 'gene', for which expression and sequence data where retrieved for both mouse and human. Ensembl gene annotations [19,20] were applied thoughout the analysis.

### Co-expressed gene sets were defined from a compendium of mouse and human expression data and tested for functional coupling

Previous results by our group and others have shown that statistical analysis of gene expression profiles in a large compendium of expression data can predict targets of differentiation processes, and identify functionally coupled genes [12,21,22]. In the following analysis, we specifically focused on a compendium derived from the recently completed Novartis expression atlas (SymAtlas) [23]. These data contain transcription profiles for 140 mouse and human tissues generated by hybridization on customized Affymetrix chips, and cover a large fraction of the mouse and human protein-coding genes. Sequence annotation of the Novartis probes linked the mouse and human data to approximately 17.000 unique Ensembl gene identities in each species (Table 1). Between the two datasets, 13282 non-redundant ortholog pairs could be identified by linkage of reciprocal Ensembl homology assignments (Table 1). In later steps, we excluded genes for which regulatory sequence could not be extracted (Methods), leaving 9561 ortholog pairs for clustering (Table 1). The final dataset included ~40% of the mouse and human Ensembl annotated genes.

**Table 1:** Gene coverage of the analysis

	**MOUSE**	**HUMAN**	**BOTH**
**Sequence data:**			
			
Ensembl genes total:	23954	21961	
Ensembl transcripts total:	34076	35685	
Ortholog pairs of Ensembl genes:			20188
Ortholog pairs with upstream sequence extracted :			13272
Ortholog pairs with upstream sequence extracted (redundancies removed):			12239 *
			
**Expression data:**			
			
Ensembl genes matching SymAtlas probes:	17552	16929	
ortholog pairs with expression data in both species:			13282 **
			
**Integrated dataset:**			
			
Two-species expression data AND regulatory sequence:			**9561**

Numbers in the 'MOUSE' and 'HUMAN' columns signify the number of unique Ensembl identifiers in each respective species. Numbers in column 'BOTH' signify ortholog pairs of Ensembl entries. The overlap between the nonredundant sequence database (*) and the nonredundant expression dataset (**) was 9561 ortholog pairs.

#### Clustering

**Figure 1:**
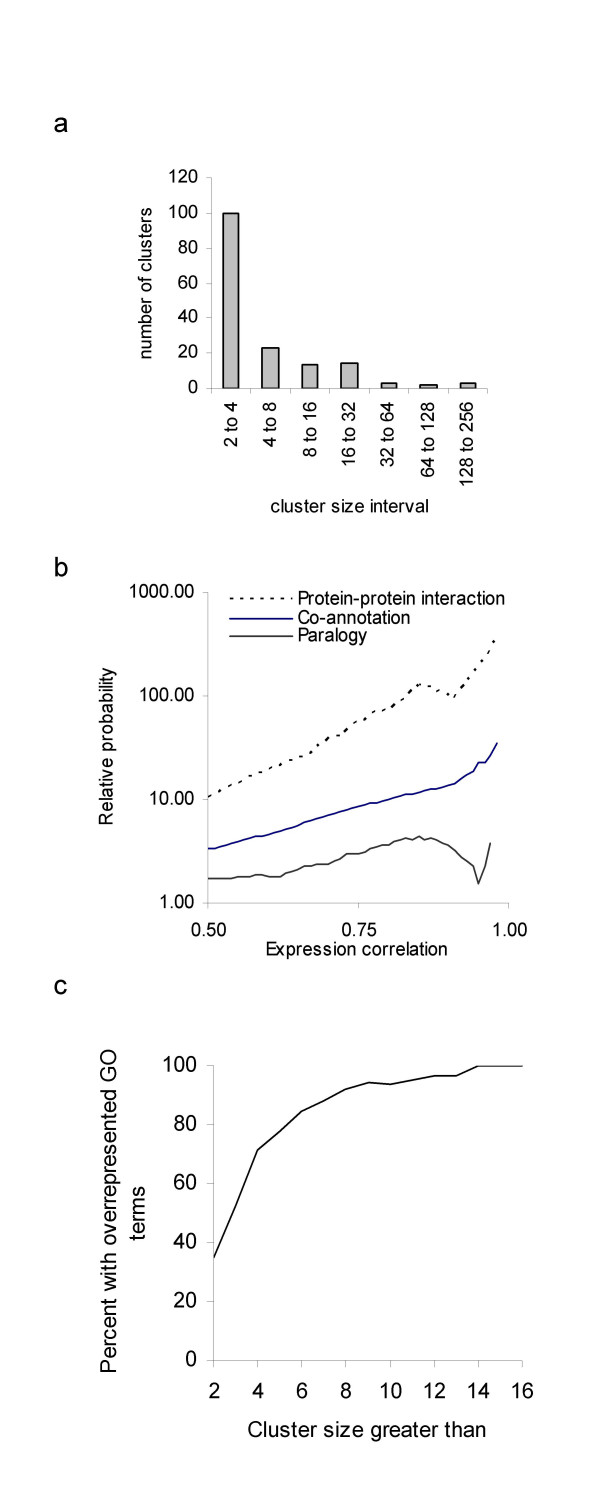
Cluster statistics A: Histogram showing the log number of clusters as a function of log cluster size, based on the clustering at Pearson correlation coefficient 0.75 cut-off. Numbers on the x axis denote cluster size intervals (2), (3–4), (5–8), (9–16),... B: Co-expression as a predictor for shared function, protein interaction and paralogy. We identified all gene pairs that correlated above or below a threshold T (X-axis). We measured the fraction of such pairs for which there was (i) a BIND database protein-protein interaction recorded in human, (ii) at least one shared gene ontology term, and (iii) evidence of paralogy. We then computed the relative probability for genes above T with this feature, compared to gene pairs below T. At expression correlation 0.80, co-expression was associated with a 100-fold relative probability for genes to encode protein interactors, a 10-fold probability for genes to share functional annotation, but only a 3-fold probability for genes to be paralogs. C: Fraction of clusters with at least one over-represented GO term (Y axis), as a function of cluster size (X axis). GO term over-representations were computed at a 10% false discovery rate.

We clustered the mouse/human ortholog pairs based on their expression profiles across the 140 mouse and human tissues (Methods). We computed clusters at cut-off levels ranging from Pearson's correlation coefficient (hereafter termed PCC) 0.61 to 0.99. At the lowest applied cut-off, 57% of all ortholog pairs in the data were members of a cluster. The cluster sizes were distributed in a skewed manner, with a predominant formation of small clusters (Figure 1A). All analyses hereafter were performed at a PCC = 0.75 cut-off, which generated 160 clusters with 2407 ortholog pairs. This relatively stringent cut-off was chosen to reduce the number of non-relevant genes in the clusters. A higher cut-off did not seem reasonable given the noise-level of the microarray experiments (as judged by the Pearson correlation between replicated samples and between probes that are annotated for the same gene, data not shown).

#### Assessment of functional linkage

According to the definition, a gene battery should encode functionally linked proteins. We used Gene Ontology (GO) terms, protein-protein interactions (from the BIND database [24]), and manual curation to assess functional linkage within our expression clusters. First, we investigated the relationship between expression profile similarity of two ortholog pairs and their relative probability to share a functional annotation term or to encode interacting proteins (Figure 1B). There was a consistent correlation between co-expression and the relative probability for two genes to share a GO-term or to encode interacting proteins (Figure 1B).

Second, we studied the statistical over-representation of GO terms and interacting proteins inside the clusters (Methods). 30/32 clusters with ten or more ortholog pairs contained at least one over-represented GO term. The proportion of small clusters with over-represented GO terms was lower, which reflects a lack of statistical power in small clusters. Genes encoding interacting protein pairs were also over-represented inside clusters. 35 cases of protein-protein interaction between two genes *in the same cluster *were found. In contrast, 1000 simulations on permuted data revealed a median of 9 interactions (observations ranging between 5 and 21). The BIND data contained only 600 interactions that could be mapped to the dataset, which explains the seemingly low number of 35 interactions.

**Figure 2:**
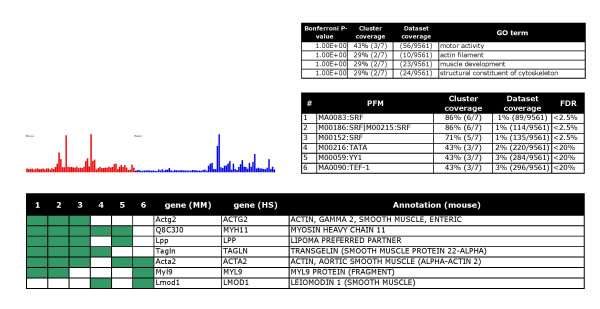
A smooth muscle differentiation battery: The bar chart (left) illustrates the average expression level of cluster members (Y axis) across arbitrarily ordered tissues (X axis) in two species (red = mouse and blue = human). Three tables list over-represented functional terms (upper small table), over represented motifs (PFMs) (middle table), and cluster members (lower table).

**Figure 3:**
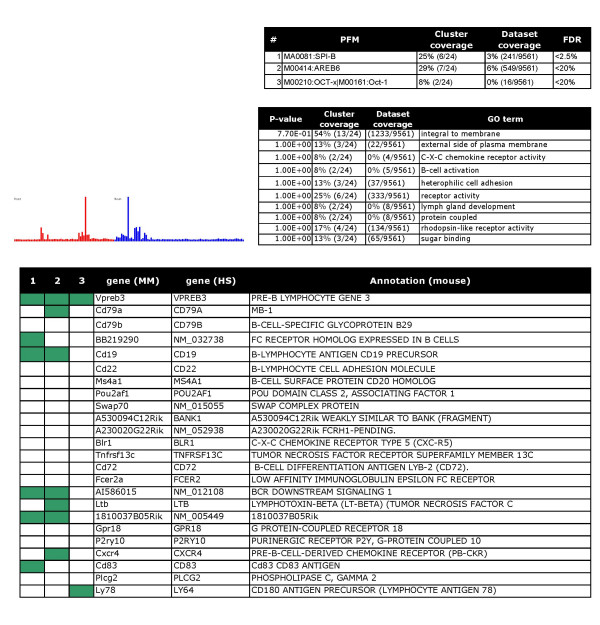
B-lymphocyte differentiation battery: Tables and charts are organized as in figure 2.

**Figure 4:**
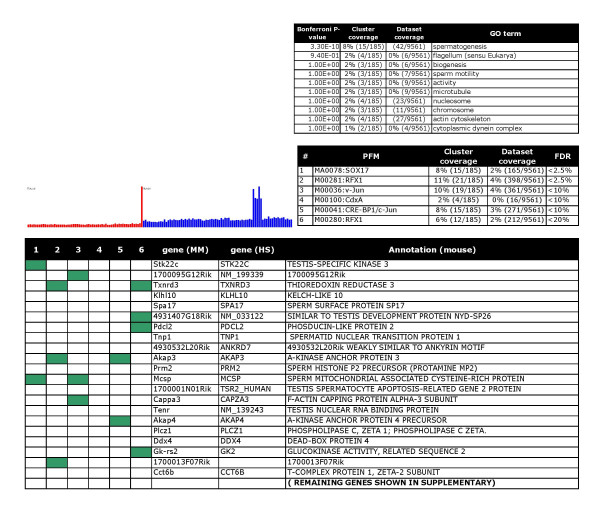
Testis selective battery: Over representation of RFX and SOX17 motifs indicates new roles for these factors as coordinators of testis selective gene expression. Tables and charts are organized as in figure 2.

**Figure 5:**
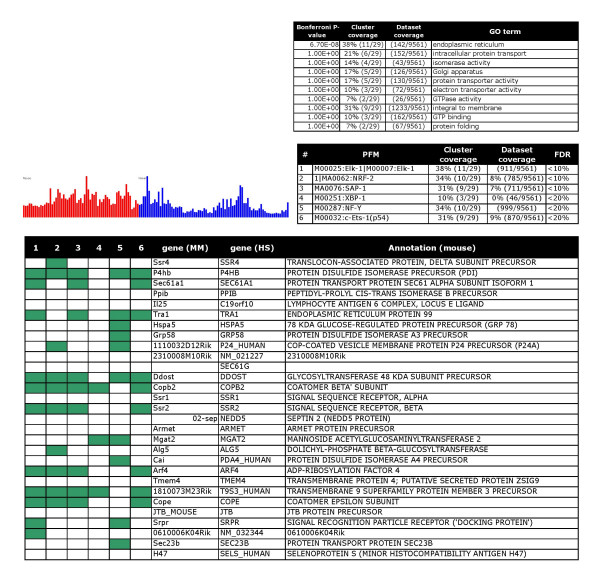
Endoplasmatic reticulum associated genes: Over representation of XBP-1, NRF and RTS motifs suggest novel functions for NRF and ETS family factors in the regulation of ER-related genes. Tables and charts are organized as in figure 2.

**Figure 6:**
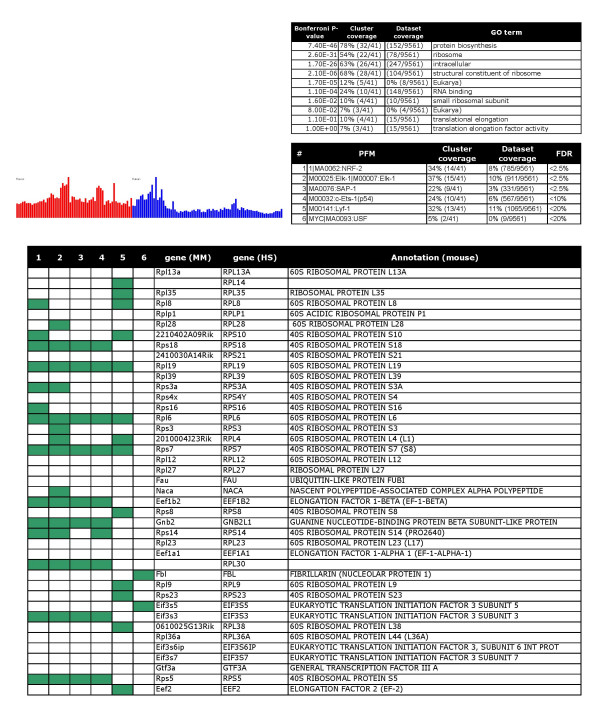
Ribosomal genes: Tables and charts are organized as in figure 2.

**Figure 7:**
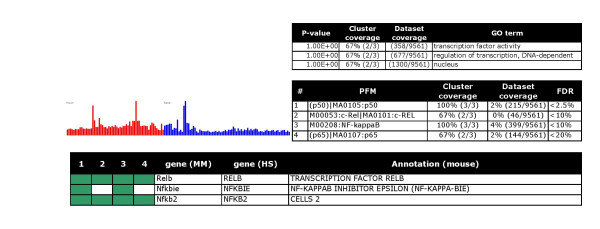
NF-kappaB pathway: Over representation of REL and NFkappaB motifs indicates feed back signalling. Tables and charts are organized as in figure 2.

Finally, the clusters were annotated by manual curation. Six examples are shown in Figures 2, 3, 4, 5, 6, 7, and clusters with predicted regulators are listed in Table 2. For a full overview, see the web supplement . Several clusters clearly represent gene sets that mediate *specialized features of different cell types*, including smooth muscle specific genes (cluster 40, Figure 2), B lymphocyte-specific genes (cluster 16, Figure 3), and genes selectively expressed in the testis (cluster 5, part shown in Figure 4). Further, we detected clusters that were related to *cellular processes or organelles*, including endoplasmatic reticulum (cluster 13, Figure 5), protein synthesis (cluster 1, Figure 6), and modulators of transcriptional regulation (cluster 65, Figure 7). A grand majority of the clusters were defined by peaks at different amplitudes in several tissues. As an example, the endoplasmatic reticulum cluster (Figure 5) was defined by a highly variant profile with strong expression in, for example, exocrine glands. Generally, the cluster profiles were conserved between species, in the sense that clusters were defined by peaks in the same organs. This effect was more pronounced for clusters with expression in a single organ, such as the testis (data not shown).

**Table 2:** over-represented motifs detected at <10% false discovery rate

**Cluster number**	**FDR**	**PFM Number**	**PFM Annotation**
			
1: Protein synthesis	<2.5%	190	M00025:Elk-1, M00007:Elk-1
	<2.5%	110	M00050:E2F, MA0024:E2F
	<2.5%	57	M00108:NRF-2, MA0028:Elk-1, MA0062:NRF-2
	<2.5%	181	MA0076:SAP-1
	<10%	18	M00074:c-Ets-1(p54)
	<10%	78	M00262:Staf
			
2: Oocyte / fertilized egg	<2.5%	71	M00024:E2F
	<2.5%	190	M00025:Elk-1, M00007:Elk-1
	<2.5%	9	M00032:c-Ets-1(p54)
	<2.5%	110	M00050:E2F, MA0024:E2F
	<2.5%	57	M00108:NRF-2, MA0028:Elk-1, MA0062:NRF-2
	<2.5%	181	MA0076:SAP-1
	<10%	238	MA0088:Staf, M00264:Staf
			
3: Neural tissues	<2.5%	99	M00189:AP-2
	<2.5%	115	M00196:Sp1
	<2.5%	141	M00256:NRSF
	<10%	75	M00243:Egr-1
			
4: Lymphocytes	<2.5%	143	MA0050:Irf-1, M00062:IRF-1, M00063:IRF-2
	<10%	74	M00054:NF-kappaB, MA0061:NF-kappaB
	<10%	28	M00258:ISRE
			
5: Testis / spermatogenesis	<2.5%	109	M00281:RFX1
	<2.5%	142	MA0078:SOX17
	<10%	108	M00036:v-Jun
	<10%	248	M00041:CRE-BP1/c-Jun
	<10%	65	M00100:CdxA
			
6: Liver	<2.5%	16	M00134:HNF-4
	<2.5%	212	M00158:COUP-TF / HNF-4, MA0017:COUP-TF
	<2.5%	33	M00206:HNF-1
	<2.5%	203	MA0046:HNF-1, M00132:HNF-1
	<2.5%	234	MA0047:HNF-3beta, M00131:HNF-3beta
	<2.5%	113	MA0065:PPARgamma-RXRal
	<10%	46	M00155:ARP-1
	<10%	212	M00158:COUP-TF / HNF-4, MA0017:COUP-TF
	<10%	146	MA0071:RORalfa-1, M00156:RORalpha1
			
8: ECM	<10%	215	M00378:Pax-4
			
9: Cardiac muscle	<2.5%	223	M00026:RSRFC4
	<2.5%	144	M00152:SRF
	<2.5%	59	M00231:MEF-2
	<2.5%	222	M00232:MEF-2
	<2.5%	161	M00252:TATA
	<2.5%	259	M00418:TGIF, M00419:MEIS1
	<2.5%	160	MA0052:MEF2
	<10%	60	M00006:MEF-2
			
12: Skeletal muscle	<2.5%	201	M00184:MyoD, M00001:MyoD
	<10%	17	M00002:E47
	<10%	59	M00231:MEF-2
			
13: Endoplasmatic reticulum	<10%	190	M00025:Elk-1, M00007:Elk-1
	<10%	57	M00108:NRF-2, MA0028:Elk-1, MA0062:NRF-2
	<10%	181	MA0076:SAP-1
			
15: Erythrocyte	<10%	209	M00128:GATA-1, M00127:GATA-1
	<10%	122	M00203:GATA-X
	<10%	198	M00413:AREB6
			
16: B lymphocyte	<2.5%	133	MA0081:SPI-B
			
17: Kidney	<2.5%	33	M00206:HNF-1
	<2.5%	188	M00411:HNF-4alpha1
			
22: Cell cycle genes	<10%	110	M00050:E2F, MA0024:E2F
			
24: Pancreas	<10%	121	M00071:E47
	<10%	193	M00080:Evi-1, M00082:Evi-1
			
30: Small intestine	<10%	31	M00346:GATA-1, M00347:GATA-1, M00348:GATA-2
			
40: Smooth muscle	<2.5%	144	M00152:SRF
	<2.5%	245	M00186:SRF, M00215:SRF
	<2.5%	88	MA0083:SRF
			
44: Retina	<2.5%	196	M00087:Ik-2
			
45: Testis (mouse signal only)	<2.5%	164	M00253:cap
			
49: Lung/endothelium (mouse signal only)	<10%	66	M00199:AP-1, M00037:NF-E2
			
65: NfkappaB signalling	<2.5%	235	M00051:NF-kappaB (p50), MA0105:p50

Over-represented motifs arranged by cluster number. FDR column: False Discovery Rate (estimated probability for the over-representation to be a spurious detection). Motifs are are shown both by their numerical identifiers (PFM number) and by their annotation (PFM annotation). In cases where a PFM is a composite based on more than one source, the components are given separated by commas. The data where generated from the PCC = 0.75 clustering, 2 kb sequence database, at 90% phylogenetic conservation.

In combination, the GO term enrichment, the protein-protein interaction, and the manual curation convincingly show that clustered genes are functionally linked.

### Regulatory DNA and descriptions of transcription factor binding sites were extracted and pre-processed

In the next part of the analysis, individual ortholog pairs were scored for transcription factor binding sites. Binding motifs were represented in the form of Position Frequency Matrices (hereafter denoted PFMs). Based on a fixed amount of upstream DNA sequence in each ortholog pair, a statistical score was computed to predict the potential for a site in the sequence to bind the factor corresponding to a PFM (described in detail in Methods). We extracted upstream + intronic sequence from the Ensembl database, in amounts of 2, 6 or 15 kb per gene (see Methods for details on boundaries). A filter was applied that removed ortholog pairs for which the transcription start differed between the two species (>1000 bp difference, see Methods). Filtering was successful for 12239 unique ortholog pairs (Table 1). In a subsequent filter, DNA sequence that was not conserved between mouse and human was removed, so-called phylogenetic footprinting. Phylogenetic footprinting was applied at different stringency, to allow the following optimization of the protocol (below). Furthermore, all exon sequence was removed from the analysis (Methods). Finally, the sequences were matched with the expression data based on annotation, the overlap being 9561 ortholog pairs (Table 1).

322 vertebrate PFMs were downloaded from the TRANSFAC and JASPAR databases [25,26]. Since the databases appeared to contain redundant or equivalent entries, highly similar PFMs were grouped and merged using single linkage hierarchical clustering and a PFM distance measure defined in [27], which reduced the number of PFMs from 322 to 266 (Methods). This step reduced the redundancy, but did not merge all identically annotated PFMs (see for example the redundant serum response factor (SRF) PFMs in Table 2, cluster 40).

### Design of a predictive scoring system for transcription factor binding

After the retrieval and preprocessing of both sequences and PFMs, all individual sequences were tested for PFM matches using the MAST software [28], a software for identifying single or multiple motifs in sequences. MAST was set to compute one p-value for each PFM with respect to each sequence (Methods). Based on the p-values obtained from the MAST software, a *composite score *was defined as the product of the p-value in the mouse and human sequences of an ortholog pair (Methods). A *composite *score close to 0 indicates that both the mouse and human promoter sequence in the ortholog pair contains sequence elements that are in very good agreement with a certain PFM.

**Figure 8:**
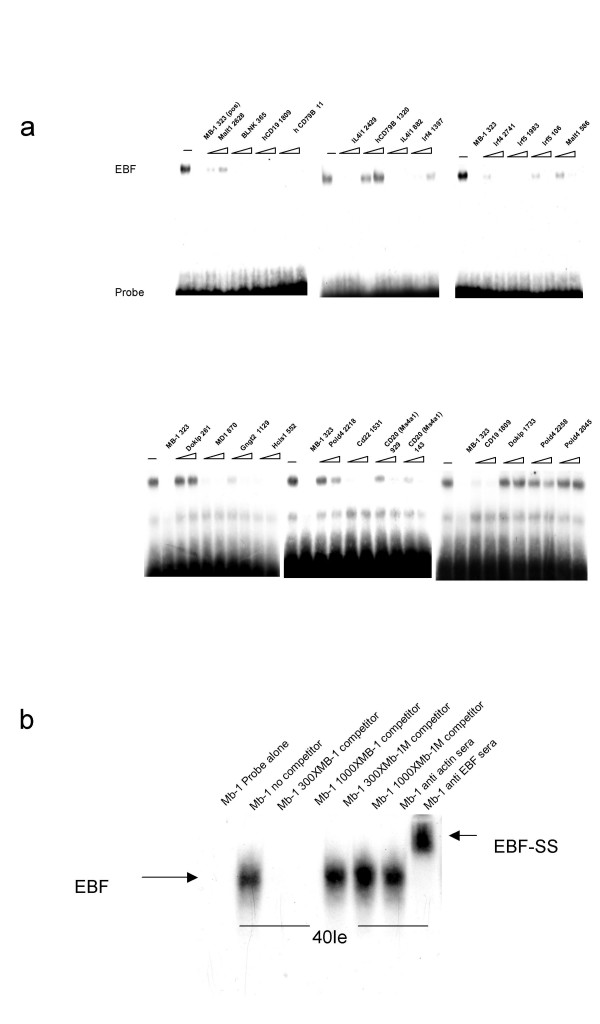
EMSA validation of EBF binding sites: A: The figure displays EMSAs in which binding of EBF to a mb-1 promoter EBF site is competed for by the inclusion of 300 or 1000-fold molar excess of unlabelled oligonucleotides that correspond to the predicted motifs. The name of the gene and the position of the motif is given in the figure. (m) indicates mouse and (h) human. "EBF" shows the position of the DNA/protein complex, and "Probe" indicates the position of free DNA. See supplementary information for a detailed description of the sites. B: The mb-1 promoter EBF site interacts specifically with EBF protein in a pre-B cell nuclear extract. The figure displays an autoradiogram in which a labelled EBF binding site from the mb-1 promoter has been incubated with nuclear extracts from 40EI pre-B cells and competitors or antibodies as indicated. EBF denotes the bound EBF protein and EBF-SS the super-shifted complex obtained by the addition of the EBF reactive antibody to the reaction mixture.

To address the biological validity of the MAST composite scores within the context of a set of co-expressed genes, we screened 48 ortholog pairs present in B-cell expressed clusters for individual EBF sites. In all, 24 individual EBF sites in 15 different ortholog pairs were detected (supplementary data, additional file 1). To test the functionality (in terms of EBF binding) of these sites, we examined the potential of 22 basepair duplex oligo-nucleotides spanning the presumed sites to compete for protein binding in Electrophoretic Mobility Shift Assays (EMSA:s). EBF binding capacity was assayed using nuclear extracts from the pre-B cell line 40-EI, a labelled mouse mb-1 promoter high affinity EBF site [29], and competitor oligo-nucleotides covering the new potential binding sites. In the absence of competitor oligo-nucleotide, a prominent DNA/protein complex (mb-1/EBF) could be detected whereas this complex was undetectable after the addition of a 300 fold molar excess of the unlabeled binding site (mb-1). The identity of the protein and the specificity of the binding were verified by competition with a point mutated mb-1/EBF site and by the inclusion of an EBF specific antibody into the reaction mixture (Figure 8B). The point mutated EBF binding site was unable to abolish complex formation even in a 1000-fold molar excess (Figure 8B), indicating that we detect specific protein DNA interactions with this experimental set up. 18 out of the 24 new binding sites competed for complex formation when added in a 300- or 1000-fold molar excess, and thus have the ability to bind EBF in vitro (Figure 8A). We conclude that the large majority of binding sites were able to functionally interact with the predicted protein, and that the composite score in principle detected factor binding. It should however be emphasized that the quality of predictions is dependent on the quality of the binding site descriptions, and the result does not necessarily imply that other predicted factors bind.

### A statistical procedure was used to detect enriched motifs in the clusters

To test whether the identified clusters represent potential gene batteries, i.e. contain shared *cis-regulatory elements*, we designed a procedure to detect significant over-representation of orthologs that match a PFM inside a cluster. The procedure is based on a modification of Fisher's exact test, which tests for dependency between two events (in this case cluster membership vs detection of a motif) [30]. We introduced a procedure to optimize the composite score thresholds for individual PFMs. In brief, we selected the threshold that gave the lowest Fisher test p-value in any one cluster. This was based on the assumption that non-random distribution of detections over clusters reflects biological function, as has been proposed in [31]. The Fisher test p-values with the optimized thresholds are hereafter termed *p-scores*.

**Figure 9:**
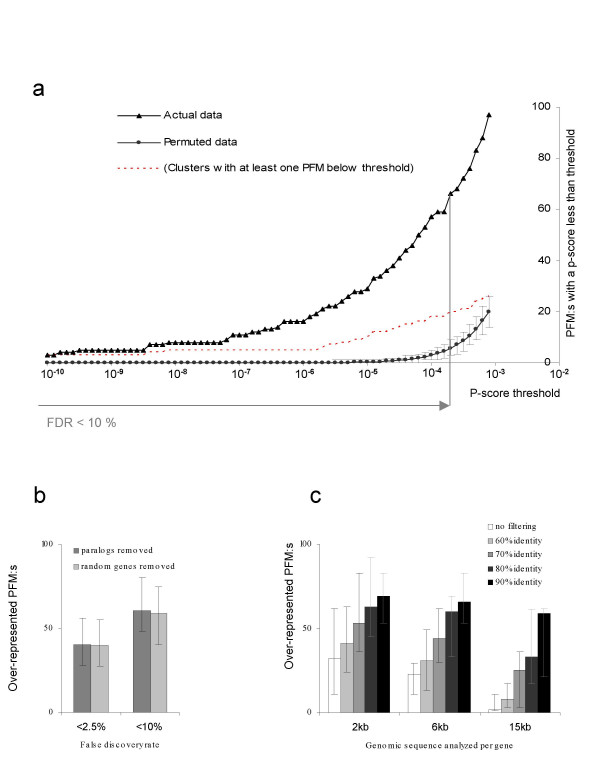
**False discovery rate estimation**. A: Illustration of how false discovery rates (FDRs) were estimated by use of simulations. Values on the y axis represent the number of motifs below a certain *p-score *(x axis). Triangles show results for observed data, filled circles show results for permuted data (error bars show the 90% confidence interval from 100 simulations). The FDR was calculated as the ratio between the simulated expectation and the observation. Red dotted line: The number of clusters with at least one enriched motif. Note that several motifs where over-represented in the same cluster. B: Removal of paralogous genes from each cluster did not affect the number of detected motifs. Consequently, co-expressed paralogs is not an important source of false positives. C: The amount of DNA used per gene, and the phylogenetic footprinting stringency has a strong effect on the number of detected over-represented motifs. The sensitivity is higher when the amount of DNA is reduced. Error bars in B and C were obtained by using the 5^th ^and 95^th ^percentiles in the simulation to define the FDR.

The tests of multiple detection thresholds, multiple clusters and 266 PFMs led to a need to compensate for mass testing. This was done by estimating false discovery rates (FDR) based on simulations on randomized data. In brief, we compared the outcome when using permuted and when using observed data at different p-scores, and defined the FDR as the ratio between the two (Figure 9A). This procedure allowed us to choose a significance threshold with a controlled expected number of spurious detections. Simulations were repeated 100 times. Since optimization of detection thresholds were repeated in each simulation round, no bias in disfavour of the control case was introduced.

### The detection of over-represented PFMs was affected by DNA amount and masking, but not affected by gene paralogy

Using the described algorithm, we analyzed the number of times a motif was over-represented when using different amounts of DNA sequence per gene (2, 6 or 15 kb) and different stringency in the phylogenetic footprinting (>0% (keep all sequence), >60%, >70%, >80% or >90% identity). The analysis was performed at 10% false discovery rate and with the clustering obtained at PCC = 0.75 clustering cut-off. At all DNA amounts, higher stringency phylogenetic footprinting appeared to be beneficial, and between DNA amounts, 2 kb and 4 kb compared favourably over 15 kb (Figure 9C). We conclude that optimal results are obtained when using a limited amount of sequence per gene (see Discussion).

A potential confounding factor in the analysis is that similarity in upstream sequence may be attributable to factors other than shared cis-regulatory elements. The most important such factor is likely to be gene paralogy, since over-represented motifs might simply represent matches to non-functional (not yet diverged) sequence in a co-expressed gene family. In total, 22343 cases of pairwise paralogy were detected in the dataset of 9561 ortholog pairs using BLAST. All genes with transcripts matches at a BLAST E-value less than 1.0 were defined as potential paralogs. Of the 2407 ortholog pairs that clustered, 219 genes had a paralog inside the same cluster. This was clearly an over-representation since filling clusters with random genes from the dataset produced an average of 122 genes with a paralog inside a cluster (values between 104 to 131 observed in 10 randomization rounds). In a side-by-side comparison, we therefore analyzed the effect of removing paralogs from a cluster before testing for over-represented motifs (see Methods), as opposed to not removing paralogs but the same number of randomly selected genes. The results showed no increase in the number of over-represented motifs when allowing paralogs in the same cluster (Figure 9B).

### 66 over-represented motifs were observed in 21 clusters

The final analysis was performed with 2 kb sequence and 80% phylogenetic footprinting (results generated with variations of these parameters are found in the web supplement). Again, the clustering generated at PCC = 0.75 was chosen for analysis. The over-representation algorithm was run in the above cases, using 100 simulations to estimate false discovery rate thresholds. Over-represented motifs with FDR:s less than 2.5% and 10% were recorded. Key features of these results are presented in Tables 2 and 3, and the complete results are available by web browser .

**Table 3:** Interpretation of over-represented motifs with respect to published evidence. (See footnote for definition of the categories.)

**I: Expected cases**		
**Number**	**Function / expression**	**Transcription factors**

4	lymphocytes	Irf1/Irf2/ISRE, NFKB [34-36]
6	liver	HNF-1 alpha and beta, HNF4 alpha [32, 59-61]
9,12	cardiac and skeletal muscle	MyoD/E47, MEF2 family factors, SRF [4, 6, 48]
15	erythroid cells	GATA-1 [62]
16	B lymphocytes	Spi-B, Oct-I* [37, 40, 63]
22	cell cycle	E2F family factors [49, 50]
40	smooth muscle	SRF [5, 64]

		
**II: Extended**		

3	neural tissue	NRSF [65]
5	testis	SOX17, RFX2 (RFX1-RFX3) [41-43]
6	liver	ARP-1/COUP-TF, PPARγ [66, 67]
13	ER	XBP-1 [68]
15,16	erythroid cells/ B cells	AREB6* [38]
17	kidney	HNF-4alpha, HNF-1-alpha/beta [69, 70]

**III: Unexpected**		

1	protein synthesis	NRF and ETS family factors
2	oocyte	E2F and ETS family factors
6	liver	ROR-alpha
8	ECM	Pax4
9	cardiac muscle	TALE family factors TGIF and MEIS1
13	ER	NRF and ETS family factors
24	pancreas	E47
30	small intestine	HNF4-alpha and GATA factors
44	retina/eye	Ik-2
45	testis	cap

Criteria for inclusion in the different groups: I. Existing evidence of co-regulation of cluster member by the factor, and/or a mutant mouse phenotype that affects differentiation of the cell type. II. Evidence of the factor acting on a limited number of genes in the tissue. Evidence of the transcription factor being expressed in the tissue. III. No or limited evidence in the literature. * = Detected at 20% FDR.

A predictive screen performed with these settings associated 66 motifs to co-expression in a total of 21 clusters at FDR thresholds 2.5% and 10% (Table 2). There was a clear tendency for motifs to be detected in most large clusters, and for smaller clusters to lack over-represented motifs. This can be directly explained by the fact that even a high degree of motif presence in a small cluster can be attributed to spurious detections, and that the Fisher test accounts for that. The over-represented motifs were validated against the literature, and fell into three main categories: ^1) ^previously described cases of gene-battery-like regulation, ^2)^previously unreported cases of gene battery-like regulation with some support in a limited number of genes, and ^3) ^hypothetical cases of gene battery regulation (Table 3, Discussion).

In the typical case, an over-represented motif did not cover all cluster members but rather a fraction. The average coverage was 15%, with observations ranging from 1% to 100%. The limited coverage can be exemplified by the relatively large (104 ortholog pairs) cluster of liver-selective genes, which contained 8 over-represented motifs at FDR<10%, where the PFM annotation implied HNF1, HNF4, ARP1, PPAR-γ, and COUP-TF as the binding factors (Table 2). These different motifs were detected in between 8 and 19 ortholog pairs, indicating a cluster coverage of less than 20%. An example of very high coverage was observed in the cluster that contained smooth muscle differentiation markers, in which 6/7 (86%) ortholog pairs were positive for a Serum Response Factor (SRF) motif. We compared our results for HNF-1 to a whole-genome experimental screen for targets of this factor in hepatocytes [32]. 28% (29/104) of the ortholog pairs inside the liver cluster contained experimentally verified HNF-1 sites, which indicates a high but not complete coverage. Cross-comparison of experimentally and computationally identified HNF-1 targets showed a 69% – 71% agreement (results for the two different HNF-1 PFMs at 2 kb/gene masked for 90% phylogenetic conservation).

As a complement to using database motifs, we applied *de novo *motif elicitation to screen the regulatory sequence of each cluster for over-represented DNA motifs that were not present in databases. A two-step procedure based on the MEME algorithm [33] was developed (Methods). The use of a cluster to define a PFM will lead to a bias when testing for over-representation. A simulation that involved motif elicitation from randomized clusters was used to account for this bias (Methods). 28 de novo motifs were identified as over-represented in relation to the null simulation (Supplementary data, additional file 1). Of these 28 motifs, 13 matched a described database motif (based on the PFM similarity score described in Methods and in footnote of supplementary table). 9/13 of the matching motifs corresponded to motifs that were over-represented in Table 3. The remaining 15 motifs may represent novel regulatory elements. These motifs, and a description of the procedure, are available in the supplementary information.

## Discussion

The gene battery theory predicts that the *core features *of differentiated cells are encoded by gene batteries, i.e. groups of functionally coupled, co-expressed genes that are regulated by similar sets of *cis-regulatory elements*. The primary aim of this article was to survey the mouse and human genomes for cases that fall within or close to the idealized gene battery concept.

Hierarchical clustering of an extensive compendium of micro-array expression data in mouse and human identified large numbers of co-expressed gene groups. A grand majority of the larger clusters were significantly enriched for genes that shared one or several GO-terms, indicating that co-expressed genes are functionally coupled. Moreover, interactions were significantly more common between proteins that are encoded by clustered pairs compared to randomly chosen pairs. 21 clusters, finally, were significantly enriched for genes that shared potential *cis-regulatory elements*

### Confirmation and extension of several cases of gene battery-like regulation

The predicted over represented motifs (Table 2) fell into three principal categories: (i) predictions in agreement with described gene batteries, (ii) predictions supported by a limited number of observations, where the analysis predicted gene battery-like regulation, and (iii) novel predictions of hypothetical gene batteries. The findings are summarized with respect to these categories in Table 3.

For type (i) predictions the method is useful for proposing new target genes. As an example, the smooth muscle cluster contains 4 validated serum response factor (SRF) targets (*Acta2*, *Actg2*, *Myl11*, *Tagln*) [5]. The method identifies two more genes in this cluster, *Myl9 *and *Lpp *as potential SRF targets in smooth muscle (Figure 2). The findings in two clusters that are relevant for lymphocytes (cluster 4 in the web supplement) and B-lymphocytes (cluster 16, Figure 3) are further examples of type (i) predictions. Cluster 4 contains genes that are expressed by various lymphocyte populations. Significantly enriched motifs were Irf1/Irf2, NF-kappaB, and ISRE. Irf1 and Ir2 appear to bind the same sites [34]. Irf1 knockout mice are impaired in their myelopoiesis [35]. NFkappaB is a well-known regulator of inflammation and immune functions [36]. Cluster 16 contains genes that are specifically expressed in B-lymphocytes. SpiB was identified as the most over-represented PFM followed by AREB6 and Oct-1. Knockout of SpiB caused specific defects in B-cell terminally differentiated functions [37]. AREB6 is a hematopoietic transcriptional repressor [38]. Whereas Oct-1 seems to be dispensable for B-cell development [39], it regulates B lymphocyte genes in combination with specific co-activators [40].

In type (ii) cases, a regulator with some degree of documentation is statistically supported to play a role in gene battery-like regulation. As an example, RFX family proteins have been shown to regulate a limited number of genes in spermatogenesis, and SOX17 has been shown to be expressed in the testis [41-43]. Here, we demonstrate that motifs corresponding to these factors are present in 8–11% of testis-selective genes (Figure 4). 29% of kidney-specific genes were covered by HNF-1 and HNF-4 sites, suggesting that these genes may be general regulators in the kidney.

Finally, type (iii) cases represent novel predictions which can be viewed as testable hypotheses of regulatory mechanisms. A complete list of PFMs in the different categories with references to the literature is presented in Table 3. Examples include a potential functional role for an Ikaros-like motif in retina-selective genes (cluster 44 in the web supplementary), and a Pax family factor acting on extracellular matrix genes with strong expression in arteries (cluster 8 in the web supplementary). Interestingly, a combination of Ets family factor motifs, Nuclear respiratory factor motifs, and E2F motifs was detected in several clusters of a housekeeping character (Table 3).

### Incomplete coverage of cis-regulatory motifs indicates that other mechanisms than co-regulation may contribute to coordinated expression

The gene battery concept predicts that upstream transcription factors coordinate the expression of target genes through binding to similar *cis-regulatory elements *in the target genes. In a recent report, Alloco and co-workers [44] studied the relationship between co-expression in yeast (as determined by correlation in a set of 610 arrays) and the probability of sharing a transcription factor (as determined by experiments). In their experiments, an expression profile correlation of 0.85 implied a probability of transcription factor sharing of >0.5. The incomplete coverage in our analysis may similarly indicate that other mechanisms than co-regulation contribute to the coordinated expression. Importantly, it is technically difficult to accurately quantify the fraction of genes in a cluster that respond to a factor. The presence of false positive gene cluster members reduces the coverage. Uncertainty in the motif assignment to ortholog pairs may also reduce the coverage. In one case study, we could use data from an independent experimental screen to confirm that the liver-selective cluster had a limited coverage of HNF-1 responders (results). Further experiments are required to resolve to what extent the lack of coverage reflects alternative regulatory mechanisms or technical limitations.

### Data limitations

The tissues in the dataset represent samples of limited morphological resolution at a fixed time point. As a consequence, co-expressed gene groups that are active under specific developmental phases, under specific environmental stresses, or in small and localized anatomical structures are likely to escape detection. Moreover, co-expressed gene groups with a peak in one tissue only are unresolved for single cell types. Low coverage of motifs in such clusters may reflect contaminating genes that derive from another cell type.

The Affymetrix technology that were used to generate the expression measurements has been validated and match results obtained with tag sampling for sufficiently abundant genes [45,46]. Low intensity signals did not correlate well, indicating that such genes are less likely to form clusters (data not shown). Cross hybridization between related genes may, in theory, contribute to correlating expression levels. We can exclude that clusters primarily form as a result of cross-hybridization, since (1) members of well-characterized gene families appear in different (the expected) clusters (eg smooth muscle and cardiac actins) and since (2) co-expressed genes tended to share GO terms or to encode interacting proteins to a higher degree than they tended to be paralogs (supplementary data, additional file 1). We cannot however exclude subtle effects related to cross-hybridization, since results with cDNA microarrays indicate this possibility [47].

Transcription factors in the same family often have similar DNA binding properties and bind to the same sites on target genes. This leads to an ambiguity in the interpretation of PFM annotations. The MEF2 motif, is for example a receptor for the mammalian MEF2A, MEF2B, MEFC and MEFD transcription factors [48]. Similarly, the E2F motif is a receptor for a family of 9 E2F family proteins that form heterodimers with another family of proteins, the DP proteins, in a way that affects the binding affinity [49]. Different E2F proteins act at different stages in the cell cycle [50], and this is not captured by our method.

One additional data limitation is that a fraction of Ensembl transcripts may lack sequence in their 5' ends. The accuracy of our transcription starts is therefore dependent on the quality of the Ensembl transcript database.

### Method considerations and perspectives

Our approach is technically related to the Toucan and ConFac tools [17,51]. Important differences and extensions are the introduction of a composite scoring system, a procedure to optimize PFM thresholds, and the use of simulation at the level of the whole clustering to measure significances.

The performance of the method was not affected by the removal of paralogous genes from the clusters (Figure 9B), and clustering of such genes is clearly not a significant source of false positive motif prediction. Consequently, we decided to include the paralogs in the final analysis since we believe that co-expression of such genes may be a result of shared *cis-regulatory elements*.

We further evaluated the effects of using different DNA amounts in the analysis. In principal, it is motivated to include a substantial amount of DNA sequence per gene, since mammalian enhancers are frequently located far upstream in relation to the transcription start, or downstream in intronic DNA. However, the benefits of including as much sequence as possible must be balanced against the risk of introducing vast amounts of non-informative sequence into the analysis. The evaluation clearly favoured using limited amounts of DNA, and high stringency phylogenetic footprinting (Figure 9C). The optimal future alternative may be to assemble a small amount of DNA from a large area of the genome using algorithms that sort out regulatory DNA regions more efficient than phylogenetic footprinting.

The approach presented here needs to be extended to generate more complete models of gene battery regulation. Modelling of *cis-regulatory element *combinations and the relative position and spacing of *elements *are examples of such extensions. The use of PCC as a measure of co-expression may need to be reconsidered if extending the approach to very large datasets, since this measure may be sensitive to noninformative signals in a majority of samples. Further, the expression levels of potential trans-regulators and co-factors (based on protein interaction networks) can be introduced. Most likely, the method will benefit from a more accurate identification of regulatory regions (Crawford-04)

## Conclusion

We screened the mouse and human genomes and transcriptomes for instances of gene battery-like regulation. Comparative clustering was highly predictive of gene function and protein interaction, which indicates that potential gene batteries could be identified this way. Based on a statistical composite score for motifs in ortholog pairs, and a simulation approach to determine significance levels, we found 21 instances of statistically supported gene battery-like regulation that were conserved between mouse and human. These included known cases of gene battery regulation in tissues such as muscle, lymphocytes, erythrocytes, and liver. A second category of predictions included regulators with some degree of documentation, e.g. in testis, kidney, and endoplasmatic reticulum. Finally, new candidate gene batteries with statistically enriched *cis-regulatory *motifs were listed.

The results of this investigation emphasizes the need to study differentiation in terms of larger transcriptional units, and extends the methodology for doing this.

## Methods

### Annotation and preprocessing of gene expression datasets

Target sequences for the Novartis Gene Atlas V2 mouse and human expression datasets [23] were matched against the Ensembl [20] collection of mouse and human transcripts using BLAST [52]. In cases where the E-value exceeded 10^-20^, the BLAST search was re-done against Ensembl gene sequences. If there was no match below 10^-20^, the probe set was excluded from further analysis. The resulting datasets covered 17552 mouse and 16929 human unique Ensembl genes (Table 1). Mouse/human orthologous gene pairs were formed using Ensembl homology maps. Redundant occurrences of the same gene in more than one ortholog pair were avoided according to the following procedure: the Ensembl human orthologs for each Ensembl mouse gene were identified. When more than one ortholog was assigned to a mouse gene, the one with the lowest positional disagreement *d *(defined below) was chosen. The procedure was repeated for all Ensembl human genes. Reciprocally matching ortholog pairs were identified, and others were excluded from further analysis. The mouse and human expression profiles of each ortholog pair were normalized with respect to mean and standard deviation and combined into a single larger profile, finally yielding an expression dataset with 13282 non-redundant ortholog pairs (Table 1).

### Preparation of upstream DNA sequence

For each ortholog pair, mouse and human candidate regulatory sequence was extracted from Ensembl. The sequence extraction algorithm starts with an ortholog pair, localizes the 5' end of the transcript in the genome in each species, and computes a value that measures the positional disagreement between the transcript 5' ends in the two species. If the disagreement is too large, the ortholog pair is excluded from the analysis. Of the 12239 ortholog pairs in the expression dataset, 9561 satisfied this criterion (Table 1). A full description of the sequence extraction procedure is available in the online supplement.

Three different lengths of DNA were extracted: 2 kb, 6 kb and 15 kb. The 2 kb dataset contained nucleotide positions ranging from -2000 to -1 relative to the transcription start, the 6 kb dataset contained positions -4000 to +2000 and the 15 k dataset contained positions -10000 to +5000.

#### Phylogenetic footprinting

The sequence datasets were subjected to phylogenetic footprinting, i.e. removal of poorly conserved sequence. The mouse and human sequences of each ortholog pair were aligned by use of the LAGAN software [53] (standard settings). Similarity was defined as the number of identical nucleotides in a 20 bp window. Nucleotides in windows with similarity below the threshold were removed. In all, five different similarity threshold were applied: >0% (no footprinting – use all sequence), >60%, >70%, >80% and >90% identity.

#### Removal of exonic sequence

Each candidate regulatory sequence was aligned to the corresponding transcripts (pairwise BLAST, e-value threshold 0.01). Nucleotides aligning with one or more transcripts were removed.

### Clustering

The set of 9561 orthologs pairs, for which both regulatory sequence and expression data could be assembled, were clustered with respect to expression pattern using hierarchical clustering [54] with average group linkage and Pearson's correlation coefficient as distance measure. In the average group linkage algorithm, cluster distances are defined as the distances between cluster means. We defined the cluster mean as the arithmetic mean of all cluster members. Correlation thresholds between 0.61 and 0.99 were applied in steps of 0.01.

### Assembly of 266 non-redundant motif position weight matrices

Motifs represented as position frequency matrices (PFMs) were downloaded from the TRANSFAC [55] and JASPAR [25] databases. Non-vertebrate matrices were filtered out. Highly similar matrices were grouped and merged using single linkage hierarchical clustering, reducing the number of PFMs from 322 to 266. Distances between matrices were calculated using a probabilistic method [56]. Individual positions between matrices were compared using the chi square test, and p-values for all overlapping positions were combined using the geometric mean. Sense and antisense of motifs were compared for all possible frameshifts with at least 75% overlap. Clustered motifs (score > 0.5) were added together in overlapping positions, and flanking positions were discarded. PFMs were transformed into position weight matrices (PWM:s) to make them compatible with the MAST software (see below). The value of each matrix element was calculated according to the following formula:



where *n *is the raw count from the corresponding position in the PFM, *N *is the the number of observations (sum of each position/column in the PFM),  and  are pseudocounts and *p*(*b*) is the background frequency of the corresponding nucleotide.

### Scoring of regulatory sequences for motif position weight matrices

#### Scoring of individual sequences

For each ortholog pair, both mouse and human regulatory sequences were scored for all motif PWM's using the MAST software [57]. MAST was set to compute whole-sequence p-values (*-seqp *setting), using a first order Markov chain background. The Markov chain background data where computed from unmasked genomic sequence -4000 upstream to +2000 downstream in all ortholog pairs.

#### MAST composite scores

The p-values reported by MAST for the mouse and human sequences of an ortholog pair were multiplied for each PWM. The result was treated as a composite score that reflects the overall "signal" for a certain binding site in the regulatory sequences of an ortholog pair. The composite score is a product of two p-values but should not be interpreted as a p-value, since the mouse and human sequences are highly dependent.

### Algorithms to detect over-representation of GO terms and motifs

#### GO over-representation

Clusters were evaluated for over-representation of GO terms using Fisher's exact test [30]. Due to the large number of tests (the number of clusters times the number of GO terms), the resulting p-values were corrected using the Bonferroni method [30].

#### Motif over-representation

The test was applied to the 9561 ortholog pairs with both sequence and expression data. These constituted the *population*. For each PWM, genes with MAST composite scores below a threshold were defined as *labeled *and the others as *unlabeled*. Further, each cluster was considered as a *sample *from the population. The algorithm is briefly sketched here and is available in detail in the online supplement:

##### Step 1: Compute p-scores under the null hypothesis

First, ortholog pairs were permuted across the dataset, making each cluster a random selection of genes. Second, an optimal composite score threshold was found for each PWM. This was done by computing the Fischer test p-value for over-representation of labeled ortholog pairs for all clusters k at a range of detection thresholds ranging from 10^-8 ^to 10^-3 ^in stepwise increases by a factor of 10^0.5^. The threshold chosen for each PWM was the one that gave the best p-value for that PWM in any cluster. P-values for over-representation of all motifs in all clusters were finally calculated using the optimized thresholds. We refer to this statistic as the *p-score*. This whole procedure was repeated for 100 iterations, which resulted in empirical estimates of how many over-represented motifs we could expect below a certain p-score under the null hypothesis.

##### Step 2: Compute p-scores for the observed data

This step was identical to step 1 but without permutations and repetitions.

##### Step 3. Compute the false discovery rate

After the simulation, we defined the false discovery rate (FDR) at p-score *p *as the expected number of over-represented motifs in the null simulation, divided by the corresponding value in the observed data.

### Electrophoretic mobility shift assay (EMSA)

40-EI and 230–238 cells were grown in RPMI medium supplemented with 10% FCS, 10 mM HEPES, 2 mM pyruvate, 50 μM 2-mercaptoethanol and 50 μg gentamicin per ml (complete RPMI media). STAT activation in 230–238 cells was achieved by 5 hours of incubation with 0.5 ng/ml recombinant mouse interferon gamma (Immunokontact, Germany). Nuclear extracts were prepared according to Schreiber et al. [58]. DNA probes were labelled with γ[^32^P] ATP (Amersham Biosciences, UK) by incubation with T4 polynucleotide kinase (Roche Diagnostics, Mannheim, Germany), and purified on a mini Quick Spin Oligo Column (Roche Diagnostics, Sweden). Nuclear extracts were incubated with labelled probe (20,000 cpm, 3 fmol) for 30 min at room temperature in binding buffer (10 mM HEPES [pH 7.9], 70 mM KCl, 1 mM dithiothreitol, 1 mM EDTA, 2.5 mM MgCl2, 4% Glycerol) with 0.75 μg Poly(dI/dC) (Amersham Pharmacia Biotech, UK). When EBF-binding was investigated, 1 mM ZnCl_2 _was supplemented to the mixture. The samples were separated on a 6% polyacrylamide TBE gel, which was dried and subjected to autoradiography. In the supershift experiments (Figure 8B), DNA competitors or antibodies (anti-EBF SC-15333, anti-Actin SC-1616, Santa Cruz Biotech) were added 10 min before the addition of the DNA probe. To visualize the super-shifted complex, the unbound probe was run out of the gel. The following Oligonucleotides were used for EMSA: *mb-1 *sense: 5'-AGCCACCTCTCAGGGGAATTGTGG-3'; *mb-1 *antisense: 5'-CCACAATTCCCCTGAGAGGTGGCT-3'; mutated *mb-1*sense: 5'-AGCCACCTCTCAGCCGTTTTGTGG-3'; mutated *mb-1 *antisense: 5'-CCACAAAACGGCTGAGAGGTGGCT-3';

## List of abbreviations

EBF Early B cell factor

EMSA Electrophoretic Mobility Shift Assay

GO term Gene Ontology term

PCC Pearson's correlation coefficient

PFM Position frequency matrix

PWM Position weight matrix

## Authors' contributions

The overall computational strategy applied was conceived by SN, EL and PM together with EK, PL, and ON. SN and EL performed the bulk of the analysis and the manuscript was drafted by SN, EL and PL with contributions from all authors. The experimental validation of EBF sites was conceived and performed by RM and MS. The *de novo *detection of motifs was conceived and performed by EK.

## Supplementary Material

Additional File 1 Supplementary.doc is a word file that contains all the supplementary information referred to in the text. Click here for file
