# Supplementary material for: Predictive screening for regulators of conserved functional gene modules (gene batteries) in mammals
Source: BMC Genomics. 2005 May 9;6:68. doi: 10.1186/1471-2164-6-68 (PMC1134656; doi:10.1186/1471-2164-6-68)
Supplement: Additional File 1 — Supplementary.doc is a word file that contains all the supplementary information referred to in the text. [file 1471-2164-6-68-S1.doc]

**Supplementary information**

**Contents**

**Results**

1. Access to our online material
2. Annotation of de novo motifs
3. Predicted EBF sites that were experimentally validated in Fig3
4. De novo elicitation of motifs

**Algorithms**

1. Algorithm to extract upstream sequence
2. Algorithm to estimate false discovery rates
3. MEME settings

**1. Web resource with results from the predictive screen**

Full results are made available on [www.wlab.gu.se/lindahl/genebatteries](http://www.wlab.gu.se/lindahl/genebatteries)

**2. Annotation of de novo motifs**

| **category** | **De novo motif** | **Consensus** | **Score** | **Database best match** | **Match score** | **lenght1** | **lenght2** | **Overlap** |
| --- | --- | --- | --- | --- | --- | --- | --- | --- |
| long | Cluster_6_2_1 | CAGTTAATGATTAA | 0.0000 |  |  |  |  |  |
| long | Cluster_6_1_3 | AGGTCAACATTAACC | 0.0151 |  |  |  |  |  |
| long | Cluster_44_1_1 | TCAAGGCAATTAA | 0.0176 |  |  |  |  |  |
| long | Cluster_40_3_1 | CCTTATTTGGTAA | 0.0252 | **M00186:SRF|M00215:SRF** | 0.35 | 13 | 13 | 12 |
| long | Cluster_40_1_2 | GGCCTTAAAAGGC | 0.0252 |  |  |  |  |  |
| long | Cluster_40_3_2 | CCTTTTATGGCCTGG | 0.0277 | M00216:TATA | 0.33 | 15 | 10 | 10 |
| long | Cluster_61_1_1 | GACATTAATGACAG | 0.0277 | M00269:XFD-3 | 0.33 | 14 | 14 | 13 |
| long | Cluster_31_2_1 | TCCCCTTGGCTTTCA | 0.0302 |  |  |  |  |  |
| long | Cluster_23_1_1 | TTCCCCCAGCTGGGA | 0.0302 |  |  |  |  |  |
| long | Cluster_41_1_1 | CACTTCCGGCCCT | 0.0403 |  |  |  |  |  |
| long | Cluster_9_1_1 | CGGCTATTTTTAG | 0.0403 | **M00006:MEF-2** | 0.45 | 13 | 16 | 13 |
| long | Cluster_40_1_3 | CAGAGAATGGCAG | 0.0554 |  |  |  |  |  |
| Long | Cluster_17_1_1 | AAAGTTAAACATTAA | 0.0630 | **M00206:HNF-1** | 0.32 | 15 | 17 | 13 |
| Long | Cluster_36_1_1 | TTTCACAAGGTCATA | 0.0655 |  |  |  |  |  |
| long | Cluster_6_2_2 | AATTTCAAAGTAT | 0.0907 |  |  |  |  |  |
| mid | Cluster_17_1_1 | GTTAAATATTAA | 0.0052 | **M00206:HNF-1** | 0.29 | 12 | 17 | 12 |
| mid | Cluster_44_1_1 | CAAGGCAATTAG | 0.0209 | M00206:HNF-1 | 0.32 | 12 | 17 | 10 |
| mid | Cluster_12_3_1 | CAGCTGTCCCC | 0.0262 |  |  |  |  |  |
| mid | Cluster_40_2_3 | CAGAGAATGGCA | 0.0366 |  |  |  |  |  |
| mid | Cluster_9_1_1 | GCTATTTTTAG | 0.0366 | **M00006:MEF-2** | 0.53 | 11 | 16 | 11 |
| mid | Cluster_40_1_1 | CCATATAAGG | 0.0419 | **M00186:SRF|M00215:SRF** | 0.59 | 10 | 13 | 10 |
| mid | Cluster_40_1_2 | CTGCCATTCTC | 0.0419 |  |  |  |  |  |
| mid | Cluster_4_1_3 | GGCGTGCGCCAC | 0.0419 |  |  |  |  |  |
| mid | Cluster_54_1_1 | CTGCCAATGGGA | 0.0628 |  |  |  |  |  |
| mid | Cluster_74_1_2 | CTGTGGTCTCCA | 0.0681 | M00214:SEF-1 | 0.58 | 12 | 19 | 8 |
| mid | Cluster_89_1_1 | TCCTTAGAAACC | 0.0733 | M00415:AREB6 | 0.3 | 12 | 9 | 8 |
| short | Cluster_40_1_1 | CCATAAAAG | 0.0645 | **M00186:SRF|M00215:SRF** | 0.43 | 9 | 13 | 9 |
| short | Cluster_9_1_1 | CTAAAAATA | 0.0806 | **M00006:MEF-2** | 0.85 | 9 | 16 | 9 |
|  |  |  |  |  |  |  |  |  |
| # Non-matching |  |  | 15 |  |  |  |  |  |
| # Matching (agreements with over-represented database motifs in bold) | | | 13 |  |  |  |  |  |
| # Total |  |  | 28 |  |  |  |  |  |

**3. EBF site predictions that were validated experimentally (Figure 3)**

Columns:

#: number of site. Listed as in same order as on gel.

Pos: approximate genomic position. (positional information is lost when aligning with lagan, and exact postion could be obtained with some work, but this has not been done here)

p-value: The MAST p-value for the individual site to match the EBF motif

Site: labeled red represents the sequence used as template for synthesizing

oligonucleotides.

| **Site** | **Approx. pos** | **p-value** | **Site** | **Gene symbol** |
| --- | --- | --- | --- | --- |
| 1 | ~-2628 | 3.40E-05 | NTGATAGGTACCAGTATCTTCCACCTCTTAAGGGCATAGAAATTAAAGCT | Malt |
| 2 | ~365 | 4.50E-06 | TATGTGCTCGGGCAAGTTTTACTTCTCCCTAGAGCAGGGGTGTTTGCCAG | BLNK |
| 3 | ~1809 | 8.20E-07 | TCTCTGCATTTGGTTCTGGGTCTCTTCCCAGGGGAGCTGTTCCGGTGGAA | CD19 |
| 4 | ~-11 | 1.80E-06 | GACAAAAGCCTGCCCTCCCCCAGGGTCCCCGGAGAGCTGGTGCCTCCCCT | CD79B |
| 5 | ~2429 | 3.60E-06 | CCTGAACAACAGGGAAAGGGGCCATTCCCCAGAGGACATCTACCAGATGG | Il4i1 |
| 6 | ~-1302 | 5.50E-05 | CACTCANNNNNNNNNNNNNNNGAACTCCCTGAAGAAGCCCCCNNNNNNNN | CD79B |
| 7 | ~882 | 1.40E-05 | ACAAGGTGAGAAGTGCTGCCTGTAGTCCTCAGGGAGCCTTTCCTGCTGCT | Il4i1 |
| 8 | ~1397 | 3.90E-05 | TCTANNNNNNNNNCAAAGTACCTTCTCTTGGGGGACTNCGGAGACTCTGG | Irf4 |
| 9 | ~-2741 | 3.20E-06 | NNNNNNNNNNATAACATTAGTAATCTCCTCGGGGAGTATGGAGCTCTGCC | Irf4 |
| 10 | ~1983 | 2.60E-06 | NNNNNNNNNNNNNNNCCAGGCAGCCTCTCCTGGGACTTCANNNTGACCTT | Irf5 |
| 11 | ~-106 | 4.90E-05 | NNNNNNNNNNNNTGTCTCCCCGGATTCCCAAAGGAGCCNNNNNNNNNNGA | Irf5 |
| 12 | ~-586 | 1.20E-05 | AGTCTCNGAAAAGATTTCGGTCAATTCCCTTATGAGTATCTGAATCTCTG | Malt1 |
| 13 | ~2613 | 5.90E-05 | NNNNNNNNNNNNACAGCTTAACTAGACCTGAGGGAACTGGGGACGGGGTC | Doklp |
| 14 | ~-870 | 1.20E-06 | CAACCCTTTAAANNNGCATGTGACCTCCCTGGGGAGCATACTGGGGGCCT | Doklp |
| 15 | ~-1129 | 9.40E-06 | CNNNNNNNNNNNGCCTCGGTGGGGTTCCCTGGGGAGCCTGCTGGGTTTTG | Gngt2 |
| 16 | ~552 | 1.40E-05 | NNNNNNNNNNCACAGGGACACAAATGCCCATGGGAAATGTAAGGGGACAG | Hcls1 |
| 17 | ~-2218 | 7.40E-06 | TGGACTCAGGAAGTGGGTGCCACATACCTTTGGGAACAGCCCCAGAATAT | Pold4 |
| 18 | ~1531 | 1.10E-05 | CACTCTGTTGGCACATCCTGCAAGATCCCTGGGGACTCACACAGAGNNNN | Cd22 |
| 19 | ~-929 | 1.20E-05 | TCGTCTAACCCTCAAGAGACTTGAGGCCCCAGGGAGTGGGGAGGCCTGGT | Ms4a1 (Cd20) |
| 20 | ~-143 | 5.90E-05 | GGTAGCATGAAGCATACCAGGCAGTCCCCAGGGTAATTTTTAAGAAGTGA | Ms4a1 (Cd20) |
| 21 | ~1809 | 8.20E-07 | NNTCTGTATCTAGTCTGGGGTCTCTTCCCAGGGGAGATGTTCCGGTGGAA | Cd19 |
| 22 | ~1733 | 2.40E-05 | NNNNGCCCTGAATGACAAGAGCAAGCCCCCAAGGCAGTGGTCTGAGANNN | Doklp |
| 23 | ~-2258 | 7.90E-06 | GTCTGCCCCACCCAGGCTTCCCTGCTCCCTCAGGGCTTTGTGGACTCAGG | Pold4 |
| 24 | ~-2045 | 8.80E-05 | ANNNNNNNNNNNNNCCCTTCCCAACTCTGTAGGGTCTCTNTCTCCCAGNN | Pold4 |
| pos cont | ~323 | 7.40E-06 | CCCCGACCCCACGCACNNNNNAGAGACTCAAGGGAATTGTGGCCAGCCCA | Cd79a |

4. Motif elicitation

To search for novel motifs, a two step method based on MEME (Bailey, Baker et al. 1997) was applied to the regulatory sequence from each cluster. Footprinted 2kb sequences (90% identity) from both mouse and human were used in the analysis. In the first step, MEME was used to identify several potential candidate motifs and for each such candidate, a new subset of genes from the original cluster was taken (based on a MAST search). For each such new set, MEME was run again, resulting in a list of novel motifs. The rationale for applying MEME twice was to generate “distinct” motifs (in terms of nucleotide agreement in each position), and to facilitate the detection of motifs in large clusters (since these will be divided into smaller clusters in the second step). In both steps the ZOOPS model was used and the background was modelled as a first order Markov chain. Since MEME has a tendency to find longer motifs more often than short motifs, the method was applied for three different cases; motifs of length 6 to 9 bp (short), motifs of length 10 to 12 bp (mid) and finally, motifs of length 13 to 15 (long).

The motifs found were assigned scores as follows. A MAST search was done on sequences in the cluster and the number of hits was counted. Another MAST search was done, this time on the set of all regulatory sequences and the number of hits was counted here as well. The score was then calculated as the ratio of the hits inside the cluster to those in the set of all sequences. Hence, this score is a number between zero and one, where a score close to one means many hits in the cluster and few hits in the set of all ortholog pairs.

To get a picture of the significance in the motif findings, a reference distribution was created by applying the same two-step method to random clusters. These clusters were of the same size as the real ones and the genes were sampled without replacement from the set of all pair of promoters used in the study. The scores from these random clusters was used as an empirical distribution, allowing calculation of a p-score for the motifs found in the real clusters by simply observing the rank of each motif in the empirical distribution. It should be noted that even if the p-score is calculated in a similar way as a p-value it should not be seen as one, since the assumptions of independence are very likely to be violated, both when it comes from the output of MEME and the randomization of the clusters.

**Motif searching results**

The below table lists our de novo motifs, according to category, consensus sequence, and a simulated p-value-like Score. Matches against database motifs are provided. See “Columns” below for further information.

|  |  |  |  |  |  |  |  |  |  |
| --- | --- | --- | --- | --- | --- | --- | --- | --- | --- |
|  |  |  |  |  |  |  |  |  |  |
| **category** | **De novo motif** | **Consensus** | **Score** | **Database best match** | **Match score** | **lenght1** | **lenght2** | **Overlap** |  |
| long | Cluster_6_2_1 | CAGTTAATGATTAA | 0.0000 |  |  |  |  |  |  |
| long | Cluster_6_1_3 | AGGTCAACATTAACC | 0.0151 |  |  |  |  |  |  |
| long | Cluster_44_1_1 | TCAAGGCAATTAA | 0.0176 |  |  |  |  |  |  |
| long | Cluster_40_3_1 | CCTTATTTGGTAA | 0.0252 | **M00186:SRF|M00215:SRF** | 0.35 | 13 | 13 | 12 |  |
| long | Cluster_40_1_2 | GGCCTTAAAAGGC | 0.0252 |  |  |  |  |  |  |
| long | Cluster_40_3_2 | CCTTTTATGGCCTGG | 0.0277 | M00216:TATA | 0.33 | 15 | 10 | 10 |  |
| long | Cluster_61_1_1 | GACATTAATGACAG | 0.0277 | M00269:XFD-3 | 0.33 | 14 | 14 | 13 |  |
| long | Cluster_31_2_1 | TCCCCTTGGCTTTCA | 0.0302 |  |  |  |  |  |  |
| long | Cluster_23_1_1 | TTCCCCCAGCTGGGA | 0.0302 |  |  |  |  |  |  |
| long | Cluster_41_1_1 | CACTTCCGGCCCT | 0.0403 |  |  |  |  |  |  |
| long | Cluster_9_1_1 | CGGCTATTTTTAG | 0.0403 | **M00006:MEF-2** | 0.45 | 13 | 16 | 13 |  |
| long | Cluster_40_1_3 | CAGAGAATGGCAG | 0.0554 |  |  |  |  |  |  |
| long | Cluster_17_1_1 | AAAGTTAAACATTAA | 0.0630 | **M00206:HNF-1** | 0.32 | 15 | 17 | 13 |  |
| long | Cluster_36_1_1 | TTTCACAAGGTCATA | 0.0655 |  |  |  |  |  |  |
| long | Cluster_6_2_2 | AATTTCAAAGTAT | 0.0907 |  |  |  |  |  |  |
| mid | Cluster_17_1_1 | GTTAAATATTAA | 0.0052 | **M00206:HNF-1** | 0.29 | 12 | 17 | 12 |  |
| mid | Cluster_44_1_1 | CAAGGCAATTAG | 0.0209 | M00206:HNF-1 | 0.32 | 12 | 17 | 10 |  |
| mid | Cluster_12_3_1 | CAGCTGTCCCC | 0.0262 |  |  |  |  |  |  |
| mid | Cluster_40_2_3 | CAGAGAATGGCA | 0.0366 |  |  |  |  |  |  |
| mid | Cluster_9_1_1 | GCTATTTTTAG | 0.0366 | **M00006:MEF-2** | 0.53 | 11 | 16 | 11 |  |
| mid | Cluster_40_1_1 | CCATATAAGG | 0.0419 | **M00186:SRF|M00215:SRF** | 0.59 | 10 | 13 | 10 |  |
| mid | Cluster_40_1_2 | CTGCCATTCTC | 0.0419 |  |  |  |  |  |  |
| mid | Cluster_4_1_3 | GGCGTGCGCCAC | 0.0419 |  |  |  |  |  |  |
| mid | Cluster_54_1_1 | CTGCCAATGGGA | 0.0628 |  |  |  |  |  |  |
| mid | Cluster_74_1_2 | CTGTGGTCTCCA | 0.0681 | M00214:SEF-1 | 0.58 | 12 | 19 | 8 |  |
| mid | Cluster_89_1_1 | TCCTTAGAAACC | 0.0733 | M00415:AREB6 | 0.3 | 12 | 9 | 8 |  |
| short | Cluster_40_1_1 | CCATAAAAG | 0.0645 | **M00186:SRF|M00215:SRF** | 0.43 | 9 | 13 | 9 |  |
| short | Cluster_9_1_1 | CTAAAAATA | 0.0806 | **M00006:MEF-2** | 0.85 | 9 | 16 | 9 |  |
|  |  |  |  |  |  |  |  |  |  |
| # Non-matching |  |  | 15 |  |  |  |  |  |  |
| # Matching (agreements with over-represented database motifs in bold) | | | 13 |  |  |  |  |  |  |
| # Total |  |  | 28 |  |  |  |  |  |  |
|  |  |  |  |  |  |  |  |  |  |
| Columns |  |  |  |  |  |  |  |  |  |
|  |  |  |  |  |  |  |  |  |  |
| **category:** | motif widht interval. Long =13-15, Mid=10-12, Short=6-9 | | | |  |  |  |  |  |
| **De novo motif** | Id of de novo identified motif. First number is the cluster number in the PCC=0.75 clustering | | | | |  |  |  |  |
| **Consensus** | Most frequent nucleotide in each position. | |  |  |  |  |  |  |  |
| **Score** | Statistical score of over-representation (scores lower than the 10th percentile of a null hypothesis simulation shown) | | | | | | | |  |
|  |  | See Methods for definition of scores and simulation procedure. | | | |  |  |  |  |
|  |  | The score compensates for the bias of over-detection in the original cluster (Methods) | | | | | | |  |
| **best match** | best match in the TRANSFAC and JASPAR databases | | |  |  |  |  |  |  |
| **Match score** | Zhang et als (see ref in paper) distance measure was used to map de novo motifs to TRANSFAC and JASPAR motifs | | | | | | | |  |
|  |  | distance >0.25 was considered a match | | |  |  |  |  |  |
| **length1** | length of de novo motif | |  |  |  |  |  |  |  |
| **length2** | length of database motif | |  |  |  |  |  |  |  |
| **overlap** | aligned overlap used to compute the Zhang measure | | |  |  |  |  |  |  |
|  |  |  |  |  |  |  |  |  |  |

**5. Algorithm to extract sequence**

For each ortholog pair, mouse and human candidate regulatory sequence was extracted by the following procedure. The algorithm starts with an ortholog pair, localizes the 5’ end of the transcript in the genome in each species, and computes a value, *d*, that measures the positional disagreement between the transcript 5’ end in the two species:

1. Establish sequence sets of the form

*{{g,t_1_,…,t_N_},{g’,t’_1_,…,t’_M_}},* where *g* and *g’* are an orthologous gene pair, and where *t_1_,…,t_N_* and *t’_1_,…,t’_M_* are the transcript(s) for each gene.

2. Align *g* and *g’* by pairwise BLAST (0.01 E-value cutoff). Let *G_k_* and *G’_k_* denote the starting position of the *k*:th block of ungapped locally aligning nucleotides in *g* and *g’*, respectively.

3. Align *g* and its corresponding transcripts *t_1_*,…,*t_N_*, (pairwise BLAST, E-value cutoff 0.01). Let *T_i_* denote the position of the *i*:th block of ungapped locally aligning nucleotides in *g*. Perform the same alignment for *g’* and its corresponding transcripts *t’_1_,…,t’_M_* and denote the j:th ungapped block with *T’_j_*.

4. For each sequence set, find the values of *i,j,k* that minimize *d=|(G_k_-Ti)-(G’_k_-T’j)|.*

5. If d>1000, exclude ortholog pair *g , g’* from the analysis (since the positional disagreement of transcription starts is fairly large, > 1000 bp).

6. Otherwise, include the ortholog pair *g, g’.* for the above values of *i,j* and *k*: extract a fixed number *w* of nucleotides from sequence *g*, starting at position *G_k_ - u*. Extract *w* nucleotides from sequence *g’*, starting at point *G’_k_-u*.

**6. Algorithm to estimate false discovery rate**

Procedure used to generate Table 2. (Graphical presentation on next page.)

STEP 1: compute the p-scores for the observed data

Pscores_observed = **p_scores**(PCC=0.75,composite_scores,paralogs,perm=0,mask=0);

STEP 2: compute the p-scores for the permuted data

For 100 iterations:

Pscores_null = **p_scores**(PCC=0.75,composite_scores,paralogs,perm=1,mask=0);

STEP 3: Find thresholds corresponding to FDR< 2.5% and 10%

For a range of p-score thresholds T:

Y(T) = Count the number of entries in Pscores_observed < T

Y0(T) = Count the average number of entries Pscores_null < T

FDR<2.5% cut-off value = the highest observed value of T for which Y0(T)/Y(T)<0.025

FDR<10% cut-off value = the highest observed value of T for which Y0(T)/Y(T)<0.010

STEP 4: Export table 2.

Table2 = all entries in Pscores_observed that satisfy FDR<10%.

The procedure uses a subroutine, **p_scores**, that returns a matrix where rows=cluster numbers, and columns=motif numbers. Each entry in this matrix is the Fisher p-score which was computed after threshold optimization.

The graphic on next page illustrates the procedure. Light blue box represents the p_score function, and the flow along red arrows represents the 4 steps above.

**7. MEME settings**

Motif Elicitation

Short (6-9 bp)

--------------

MEME:

Model (-mod) = ZOOPS

E-value threshold (-evt) = 10

Min width (-minw) = 6

Max width (-maxw) = 9

Number of motifs in the first step (-nmotifs) = 15

Number of motifs in the second step (-nmotifs) = 5

Other command line arguments: -dna -revcomp -text

MAST:

P-value threshold = 0.01

E-value threshold (-ev) = 1000000

Other command line arguments: -text -stdout -nostatus

Mid (10-12 bp)

--------------

MEME:

Model (-mod) = ZOOPS

E-value threshold (-evt) = 1

Min width (-minw) = 10

Max width (-maxw) = 12

Number of motifs in the first step (nmotifs) = 15

Number of motifs in the second step (nmotifs) = 5

Other command line arguments: -dna -revcomp -text

MAST:

P-value threshold = 0.01

E-value threshold (-ev) = 1000000

Other command line arguments: -text -stdout -nostatus

Long (13-15 bp)

--------------

MEME:

Model (-mod) = ZOOPS

E-value threshold (-evt) = 1

Min width (-minw) = 13

Max width (-maxw) = 15

Number of motifs in the first step (nmotifs) = 15

Number of motifs in the second step (nmotifs) = 5

Other command line arguments: -dna -revcomp -text

MAST:

P-value threshold = 0.01

E-value threshold (-ev) = 1000000

Other command line arguments: -text -stdout -nostatus

GetFasta 0.3-3 (www.math.chalmers.se/~erikkr/GetFasta) was used to

manipulate the FASTA files.

Python 2.3.3 (www.python.org) was used to parse the result files from MEME

and MAST.

Bailey, T. L., M. E. Baker, et al. (1997). "An artificial intelligence approach to motif discovery in protein sequences: application to steriod dehydrogenases." J Steroid Biochem Mol Biol **62**(1): 29-44.
